# Correlation between UGT1A1 polymorphism and efficacy and toxicity of irinotecan in Chinese cancer patients

**DOI:** 10.3389/fphar.2025.1563566

**Published:** 2025-03-18

**Authors:** Shuai Geng, Yulong Shen, Chen Zhang, Nan Wang, Xinyue Gao, Xinyu Luo, Ning Shi

**Affiliations:** ^1^ Department of Pharmacy, The Ninth Medical Center, Chinese PLA General Hospital, Beijing, China; ^2^ Department of Radiotherapy, The Ninth Medical Center, Chinese PLA General Hospital, Beijing, China; ^3^ Department of Medical Imaging, The Ninth Medical Center, Chinese PLA General Hospital, Beijing, China; ^4^ Department of Pharmacy, Harbin Medical University Cancer Hospital, Harbin, Heilongjiang, China

**Keywords:** gene polymorphism, UGT1A1∗6, UGT1A1∗28, irinotecan, efficacy, toxicity, systematic review

## Abstract

**Objective:**

To assess the association between UGT1A1*6/*28 polymorphisms and Irinotecan (IRI) efficacy/toxicity in Chinese cancer patients.

**Method:**

We systematically searched PubMed, Cochrane, CNKI, and Wanfang databases. Two investigators independently conducted literature screening, data extraction, and meta-analysis using Revman 5.4.

**Results:**

This study included 19 clinical trials or case-control studies, with a total of 1,698 patients. Meta-analysis showed that, ① There was no correlation between UGT1A1*6 or UGT1A1*28 gene polymorphism and IRI efficacy; ② UGT1A1*6 or UGT1A1*28 gene polymorphisms are associated with grade 3–4 diarrhea, grade 3–4 neutropenia, and grade 3–4 leukopenia, and the above-mentioned toxic reactions are more common in wild types (GG and TA6/6). ③ There was no correlation between UGT1A1*6 and UGT1A1*28 mutations and the efficacy of IRI; ④ The double wild type was more prone to grade 0–2 neutropenia, the single-site variant was more prone to grade 0–2 diarrhea, and the double-site variant was more prone to grade 3–4 neutropenia, but none of them were related to leukopenia.

**Conclusion:**

UGT1A1*6/*28 polymorphisms predict IRI-induced toxicity severity but not therapeutic efficacy in Chinese patients. These variants may serve as predictive biomarkers for personalized IRI chemotherapy.

## Introduction

Irinotecan (IRI), a semi-synthetic water-soluble camptothecin derivative, exerts its antitumor activity through its active metabolite SN-38 (7-ethyl-10-hydroxycamptothecin), a potent DNA topoisomerase I inhibitor. By stabilizing the DNA-topoisomerase I complex during replication, SN-38 induces persistent DNA single-strand breaks, thereby blocking DNA replication and suppressing RNA synthesis, ultimately leading to tumor cell death (De et al., 2018). As a first-line chemotherapeutic agent, IRI is extensively used in treating advanced solid malignancies, including colorectal cancer, gastric cancer, and small cell lung cancer, particularly demonstrating survival benefits in gastrointestinal tumors. However, its clinical utility is significantly hampered by dose-limiting toxicities: delayed-onset diarrhea (46% incidence) and neutropenia (30% incidence), both associated with life-threatening complications and treatment discontinuation rates exceeding 50% in severe cases (Paulík et al., 2020).

The metabolic detoxification of IRI is primarily mediated by uridine diphosphate glucuronosyltransferase 1A1 (UGT1A1), which catalyzes the glucuronidation of SN-38 to its inactive form SN-38G. Genetic polymorphisms in UGT1A1 profoundly influence this metabolic pathway, with two key variants—UGT1A128 (characterized by a TA-repeat polymorphism in the promoter region) and UGT1A16 (a missense mutation in exon 1)—showing ethnic-specific distributions and functional impacts. These polymorphisms reduce UGT1A1 enzymatic activity by 30%–70%, leading to SN-38 accumulation and elevated toxicity risks. Notably, UGT1A128 is more prevalent in Caucasian populations (30%–40%), whereas UGT1A16 predominates in Asian cohorts (15%–20%) (Nelson et al., 2021; Takano and Sugiyama, 2017; Dean, 2018; Deng, 2021). While substantial evidence supports the role of UGT1A1 variants in predicting IRI-induced hematologic and gastrointestinal toxicity, their association with therapeutic efficacy remains controversial. Some studies suggest genotype-dependent differences in tumor response rates, whereas others, including a pivotal analysis by (Matsuoka and Ando, 2015), found no significant correlation between UGT1A1 polymorphisms and clinical outcomes. This discrepancy underscores the need for population-specific investigations to clarify the dual predictive potential of these genetic markers.

To address this knowledge gap in Chinese patients, we conducted a systematic meta-analysis evaluating the impact of UGT1A16 and UGT1A128 polymorphisms on both toxicity profiles and treatment efficacy of IRI-based regimens. Our findings aim to optimize genotype-guided dosing strategies and advance precision oncology practices in China.

## Materials and methods

### Search strategy and study selection

To comprehensively and thoroughly investigate the impact of UGT1A16 and UGT1A128 gene polymorphisms on the efficacy and adverse reactions of irinotecan (Irinotecan, IRI) in cancer treatment, we employed a comprehensive literature search strategy. Firstly, leveraging advanced computer technology, we systematically searched multiple authoritative Chinese and English databases, including PubMed, Cochrane Library, CNKI (China National Knowledge Infrastructure), and Wanfang Data. During the search process, we combined the use of subject headings and free-text terms to ensure the comprehensiveness and accuracy of the search results. Subject headings allowed for precise targeting of research in the relevant fields, while free-text terms captured a broader range of literature potentially related to the topic, enhancing search efficiency when used together.

In terms of Chinese search terms, we meticulously selected vocabulary closely related to the topic, including irinotecan, gene polymorphism, uridine diphosphate glucuronosyltransferase 1A1 (UGT1A1), tumor, colon cancer, rectal cancer, gastric cancer, small cell lung cancer (SCLC), clinical trial, and randomized controlled trial. These terms comprehensively covered our research interests, ensuring the completeness of the search results.

For English search terms, we adopted corresponding English vocabulary matching the Chinese terms, including Irinotecan, IRI, gene polymorphism, UGT1A16, UGT1A128, colon cancer, rectal cancer, gastric cancer, SCLC, clinical trial, and randomized controlled trial. The use of these English terms enabled us to access the latest research findings on this topic internationally.

The search time frame covered from the inception of each database to December 2023, ensuring that we captured all potential relevant studies. Furthermore, to further improve the recall rate of the search, we manually reviewed the references cited within the literature. This approach allowed us to discover important literature that might have been overlooked by database search strategies, thereby enriching our research materials.

By combining multiple strategies, we ensured a comprehensive and in-depth exploration of the impact of UGT1A16 and UGT1A128 gene polymorphisms on the efficacy and adverse reactions of irinotecan in cancer treatment. These efforts will provide us with more accurate and comprehensive research evidence, contributing to the advancement of research in this field.

We specify exclusion and inclusion criteria in advance. *Inclusion criteria:* (1) Study type: clinical trial or randomized controlled trial based on UGT1A1 gene polymorphism and IRI efficacy and adverse reactions; (2) Study population: Chinese population; (3) Subjects: Patients diagnosed as cancer by pathology or cytology; (4) UGT1A1 gene polymorphism: UGT1A1*6 and UGT1A1*28; (5) Outcome indicators: The disease remission rate (RR = CR + PR) and toxic reactions (diarrhea, neutropenia, leukopenia, thrombocytopenia and hemoglobinemia) were analyzed in recessive gene model (i.e., wild type VS mutant). *Exclusion criteria* (1) Review, meta-analysis, animal experiments, case reports, summaries, editorials and letters to editors. (2) Repeated published literature; (3) Non-Chinese and English literature; (4) There is no reference data; (5) Single cohort non-comparative study.

### Data extraction and quality assessment

The two evaluators independently utilized literature management tools to conduct double-blind screening, ensuring that neither evaluator could view the other’s results until all screenings were completed independently. A structured screening form was designed, mandating the completion of inclusion/exclusion rationales item by item to minimize subjective judgment bias. Additionally, consistency was reported separately for the title/abstract screening and full-text screening phases to demonstrate rigor. In cases of disagreement, the two evaluators conducted a second independent review of the disputed articles, with a focus on verifying whether the original screening criteria aligned with the predefined standards. If disagreements persisted, a consensus meeting was convened to discuss each item individually and invoke the terms of the research protocol to reach an agreement. Alternatively, a third senior researcher (typically the project leader or methodological expert) was introduced to conduct an independent assessment, with their decision serving as the final outcome. The contents of data extraction mainly include: (1) The first author/published year; (2) Basic characteristics of the subjects: age, sex, etc.,; (3) Sample size; (4) The method of genotyping, UGT1A1*6 and UGT1A1*28 polymorphism; (5) Curative effect and toxicity outcome index. For the original study with multiple groups, the data related to this study are extracted.

The Newcastle-Ottawa scale (NOS) was used to evaluate the quality of the literature (Lazarus et al., 2019), and the quality of the included studies was evaluated according to the following eight criteria: (1) Representativeness of the exposure cohort; (2) Selection of non-exposed queues; (3) Determination of exposure method; (4) No outcome events occurred before the study began; (5) Comparability between exposed queues and non-exposed queues; (6) Evaluation of outcome events; (7) Whether the follow-up time is long enough; (8) Whether the follow-up is complete. Documents rated 7-9 are considered “high” in quality, 4–6 as “average” and 3 or less as “low”. The quality evaluation is carried out independently by two researchers and cross-checked. If there is any difference, please ask the third researcher to help solve it.

### Statistical analysis

RevMan5.4 software was used for Meta-analysis. The correlation between UGT1A1*6 and UGT1A1*28 gene polymorphism and the efficacy and adverse reactions of IRI was evaluated by odds ratio (OR), relative risk (RR) and 95% CI. Heterogeneity included in the study results was analyzed by χ^2^ test (the test level was α = 0.1), and the size of heterogeneity was quantitatively judged by combining I^2^. When *P* > 0.1 and I^2^ < 50%, it indicates that there is no statistical heterogeneity in each RCTs, and a fixed-effect model is used; On the contrary, the random effects model was used under the premise of excluding clinical heterogeneity. Significant clinical heterogeneity was treated by subgroup analysis or sensitivity analysis, or only descriptive analysis. According to the recommendation of Cochrane Systematic Review Production Manual, when the number of included literatures is ≥10, the publication bias test is carried out by funnel diagram.

## Results

### Basic characteristics and quality evaluation of literature

**FIGURE 1:**
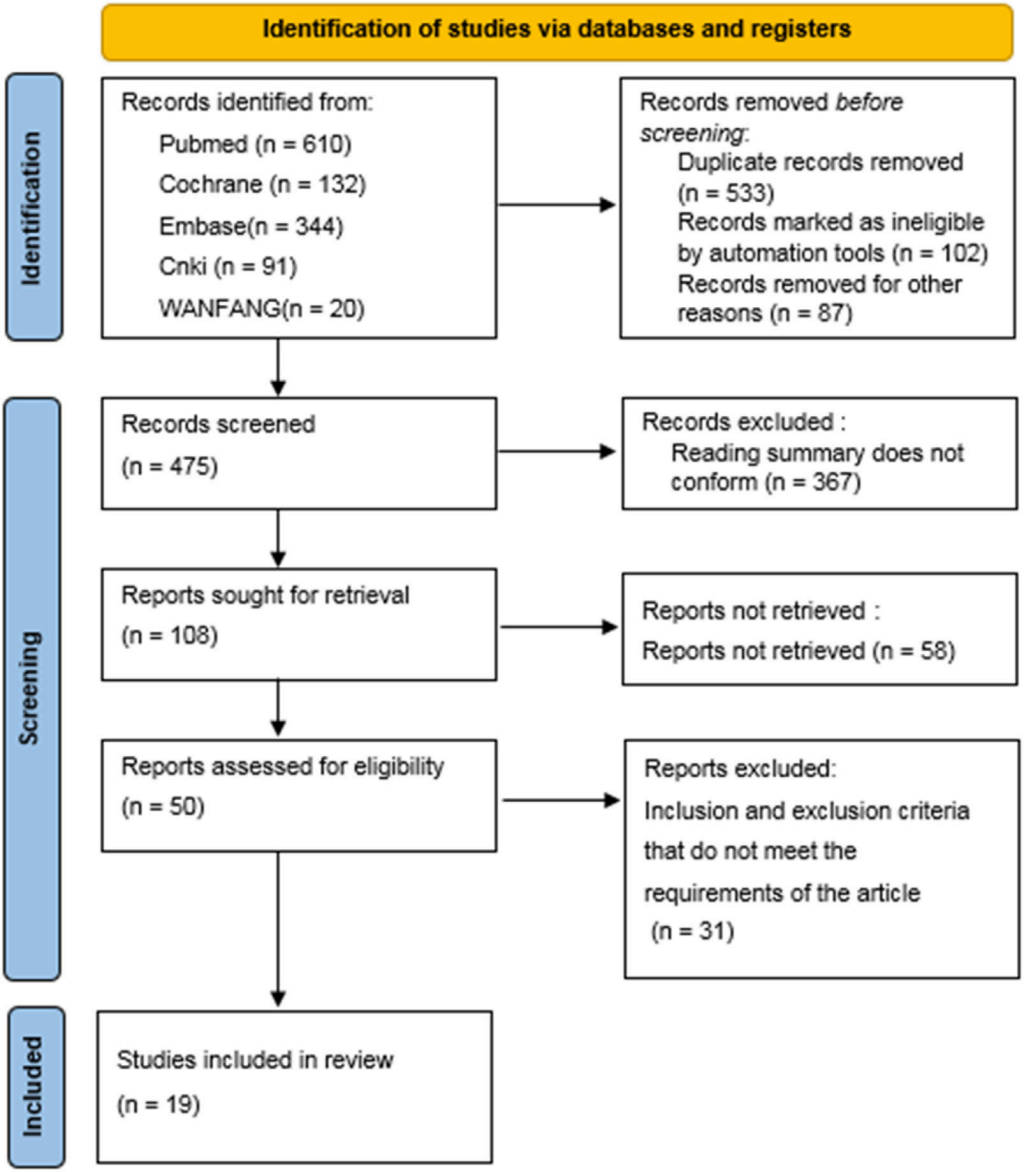
Flow chart of literature search.

1,197 related literatures were initially detected, including PubMed (n = 610), Embase (n = 344), The CochraneLibrary (n = 132), CNKI (n = 91) and Wanfang Database (n = 20). 533 duplicate literatures were eliminated by EndNote software, and 645 were excluded after reading. Finally, 19 literatures were included (Yin and Gao, 2014; Chen et al., 2018; Chen et al., 2017a; Pan et al., 2017; Wu et al., 2013; Zhang et al., 2012; Yang et al., 2017; Ji et al., 2010; Zhang et al., 2014; Jia and Fu, 2019; Zhang et al., 2019; Lv et al., 2012; Wang et al., 2018; Yan et al., 2012; Hou et al., 2023; Hua et al., 2019; Bai et al., 2017; Li et al., 2014; Xu et al., 2016) and 1,698 cancer patients were included. The retrieval process, the basic characteristics of the research and the results of bias risk assessment are shown in Figure 1 and Table 1.

**TABLE 1:** Characteristics and methodological quality of involved studies [9–27].

Involved studies	Year	Region	Cancer types	Number	Gender (Male/Female)	Age	Sample	NOS
Yin H [9]	2014	Suzhou	Gastrointestinal tumor	68	46/22	34–78	Peripheral blood	7
Chen SJ [10]	2018	Guangxi	Metastatic colorectal cancer	86	54/32	21–78	Peripheral blood	7
Chen R [11]	2017	Xinjiang	Advanced colorectal cancer	62	38/24	33–63	Peripheral blood	7
Pan RY [12]	2017	Jiangsu	Advanced colorectal cancer	60	37/23		Peripheral blood	7
Wu Q [13]	2013	Anhui	Metastatic colorectal cancer	38	27/11	25–77	Peripheral blood	7
Zhagn XJ [14]	2012	Guangzhou	Metastatic colorectal cancer	56	35/21	21–78	Peripheral blood	7
Yang MD [15]	2016	Liaoning	Colorectal cancer	65	---	---	Peripheral blood	7
Ji CS [16]	2010	Anhui	Advanced colorectal cancer	64	42/22	---	Peripheral blood	7
Zhang Y [17]	2014	Beijing	Advanced colorectal cancer	102	67/35	24–74	Peripheral blood	7
Jia XY [18]	2019	Wuhan	Colorectal cancer	110	---	18–75	Peripheral blood	7
Zhang CL [19]	2019	Shanghai	Metastatic colorectal cancer	38	25/13	45–79	Peripheral blood	7
Lu YL [20]	2012	Hebei	Gastrointestinal tumor	57	34/23	21–74	Peripheral blood	7
Wang Q [21]	2018	chekiang	Colorectal cancer	72	38/34	24–75	Peripheral blood	7
Wang Y [22]	2012	Beijing	Advanced colorectal cancer	192	114/78	26–81	Peripheral blood	7
Hou WJ [23]	2023	Nanjing	Small cell lung cancer	77	64/13	53–63	Peripheral blood	7
Hua L [24]	2019	Guangxi	Small cell lung cancer	120	72/48	19–72	Peripheral blood	7
Yu Bai [25]	2017	Beijing	Lung cancer, esophageal cancer, colon cancer	81	67/14	28–79	Peripheral blood	7
Minmin Li [26]	2014	Shandong	Metastatic colorectal cancer	167	87/80	27–71	Peripheral blood	7
Chunlei Xu [27]	2016	Xinjiang	Advanced colorectal cancer	183	124/59	---	Peripheral blood	7

## Meta-analysis results

### Correlation of UGT1A1*6 polymorphism with efficacy and toxicity of IRI

#### Efficacy evaluation

**FIGURE 2:**
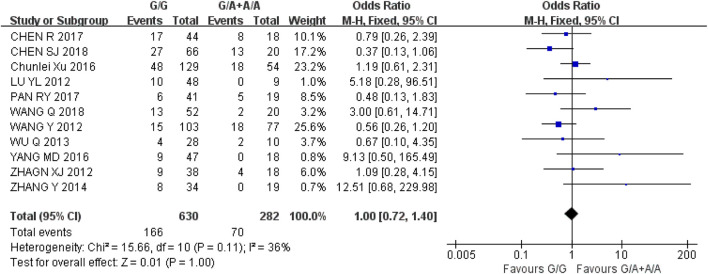
Forest map of Meta-analysis results of UGT1A1*6 and RR.

According to the evaluation standard of curative effect of solid tumor (RECIST 1.1), it can be divided into complete response (CR), partial response (PR), stable disease (SD) and progressive disease (PD). We selected disease remission rate (RR = CR + PR) to evaluate the relationship between UGT1A1*6 gene polymorphism and the efficacy of IRI. A total of 11 studies and 912 cancer patients were included. We used a recessive gene model (GG vs. GA + AA) to analyze the included studies. Heterogeneity analysis showed that I^2^ = 36%, *P* = 0.11, and there was no statistical heterogeneity among the studies. Fixed effect model was used for analysis. The results showed that there was no significant difference in disease remission rate [OR = 1.00, 95% CI (0.72, 1.40), *P* = 1.00] between wild type (GG) and mutant type (GA + AA) in recessive gene model (*P* > 0.05), as shown in Figure 2. It was suggested that there is no correlation between UGT1A1*6 gene polymorphism and IRI curative effect.

### Toxicity

**FIGURE 3:**
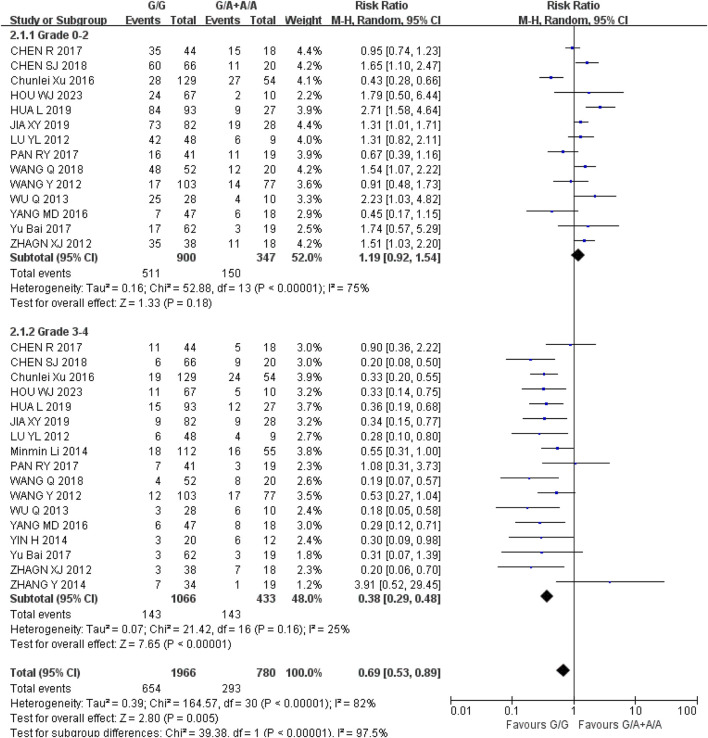
Forest map of Meta-analysis results of the correlation between UGT1A1*6 polymorphism and the incidence of diarrhea.

*Diarrhea* (1) Grade 0–2: Recessive gene model (GG vs. GA + AA) was included in 14 studies. Heterogeneity analysis showed that I^2^ = 75%, *P* < 0.00001, and there was statistical heterogeneity among the studies. The results showed that the incidence of grade 0–2 diarrhea (RR = 1.19, 95% CI (0.92, 1.54), *P* = 0.18) was not significantly different between wild type (GG) and mutant type (GA + AA) of UGT1A1*6 (*P* > 0.05); (2) Grade 3–4: Recessive gene model (GG vs. GA + AA) was included in 17 studies. Heterogeneity analysis showed that I^2^ = 25%, *P* = 0.16, and there was no statistical heterogeneity among the studies. The results showed that the incidence of 3-4 diarrhea in UGT1A1*6 wild type (GG) was lower than that in mutant type (GA + AA) during IRI chemotherapy, and the difference was statistically significant [RR = 0.38, 95% CI (0.29, 0.48), *P* < 0.00001], as shown in Figure 3. Therefore, UGT1A1*6 gene polymorphism is correlated with the incidence of grade 3–4 diarrhea in cancer patients during IRI chemotherapy, and the incidence of wild type (GG) is lower.

**FIGURE 4:**
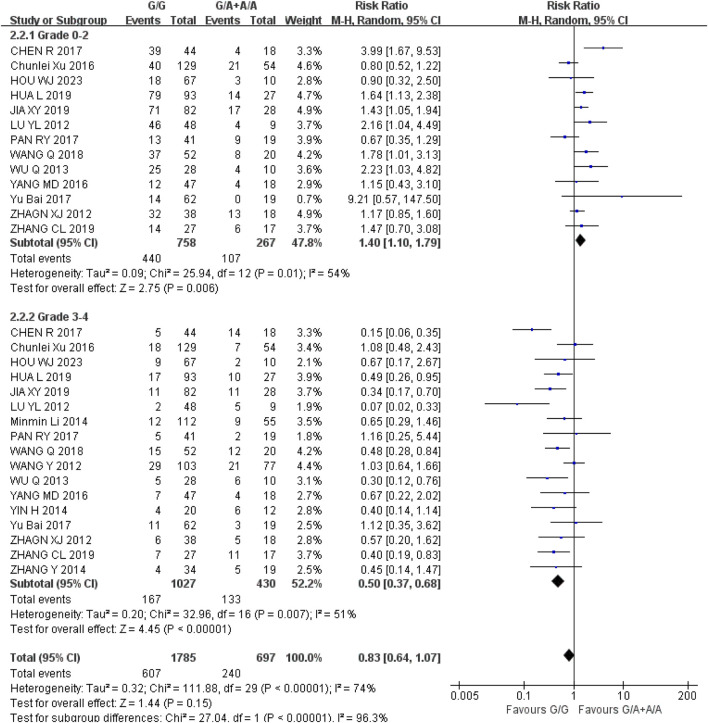
Forest map of Meta-analysis results of the correlation between UGT1A1*6 polymorphism and the incidence of granulocytopenia.

*Neutropenia* (1) Grade 0–2: The recessive gene model (GG vs. GA + AA) was included in 13 studies. Heterogeneity analysis showed that I^2^ = 54%, *P* = 0.01, and there was statistical heterogeneity among the studies. The results showed that the incidence of grade 0–2 neutropenia in UGT1A1*6 wild type (GG) was higher than that in mutant type (GA + AA) during IRI chemotherapy, and the difference was statistically significant [RR = 1.40, 95% CI (1.10, 1.79), *P* = 0.006]; (2) Grade 3–4: The recessive gene model (GG vs. GA + AA) was included in 17 studies, and the heterogeneity analysis showed that its I^2^ = 51%, *P* = 0.007, and there was statistical heterogeneity among the studies. The results showed that the incidence of 3-4 neutropenia in cancer treated with IRI was lower in wild type GG than in mutant type (GA + AA), and the difference was statistically significant [RR = 0.50, 95% CI (0.37, 0.68), *P* < 0.00001], as shown in Figure 4. Therefore, UGT1A1*6 gene polymorphism is correlated with the incidence of neutropenia in cancer patients during IRI chemotherapy, and the incidence of mild neutropenia is higher in wild type (GG) and severe neutropenia is higher in mutant type (GA + AA).

**FIGURE 5:**
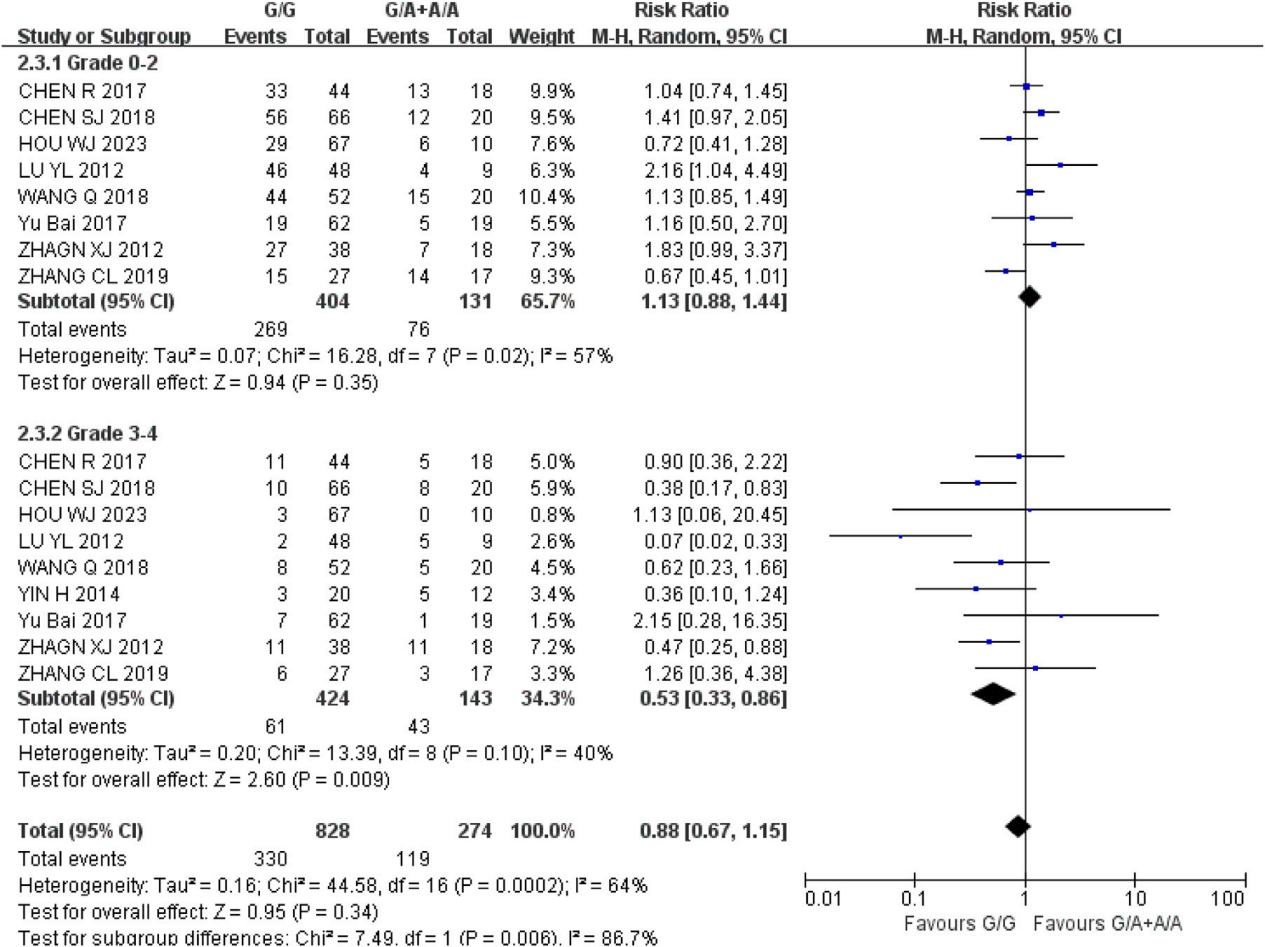
Forest map of Meta-analysis results of the correlation between UGT1A1*6 polymorphism and the incidence of leukopenia.

*Leukopenia* (1) Grade 0–2: Recessive gene model (GG vs. GA + AA) was included in eight studies. Heterogeneity analysis showed that I^2^ = 57%, *P* = 0.02, and there was statistical heterogeneity among the studies. The results showed that there was no significant difference in the incidence of grade 0–2 leukopenia between wild type (GG) and mutant type (GA + AA) of UGT1A1*6 during IRI chemotherapy [RR = 1.13, 95% CI (0.88, 1.44), *P* = 0.35]; (2) Grade 3–4: Recessive gene model (GG vs. GA + AA) was included in nine studies. Heterogeneity analysis showed that I^2^ = 40%, P = 0.10, and there was no statistical heterogeneity among the studies. The results showed that the incidence of grade 3–4 leukopenia in UGT1A1*6 wild type (GG) was lower than that in mutant type (GA + AA) during IRI chemotherapy for cancer, and the difference was statistically significant [RR = 0.53, 95% CI (0.33, 0.86), *P* = 0.009], as shown in Figure 5. Therefore, UGT1A1*6 gene polymorphism is correlated with the incidence of grade 3–4 leukopenia in cancer patients during IRI chemotherapy, and the incidence of wild type (GG) is lower.

**FIGURE 6:**
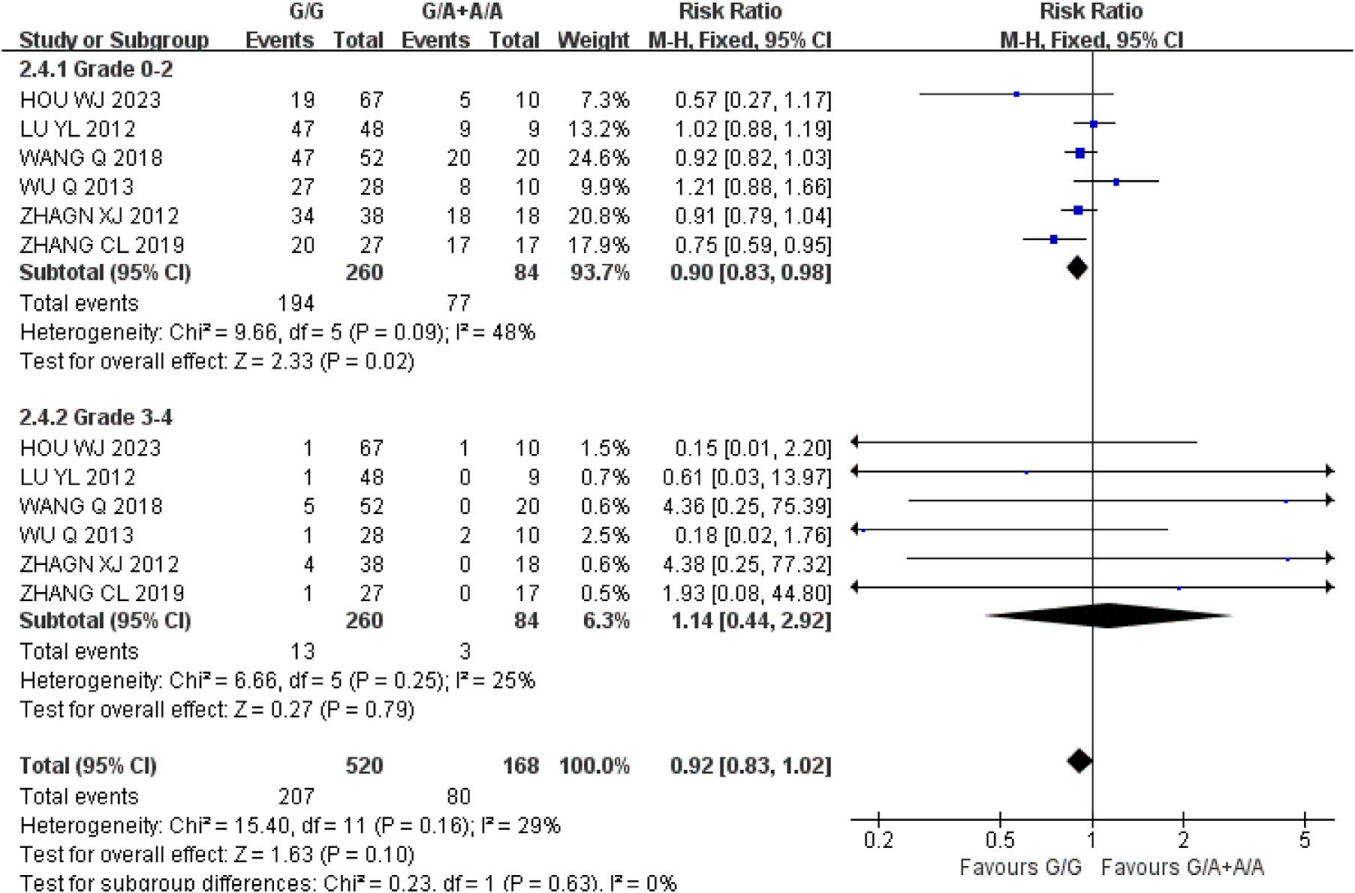
Forest map of Meta-analysis results of the correlation between UGT1A1*6 polymorphism and the incidence of thrombocytopenia.

*Thrombocytopenia* (1) Grade 0–2: A total of six studies were included in the recessive gene model (GG vs. GA + AA), and the heterogeneity analysis showed that I^2^ = 48%, *P* = 0.09, and there was no statistical heterogeneity among the studies. The results showed that the incidence of grade 0–2 thrombocytopenia in the course of IRI chemotherapy was lower in UGT1A1*6 wild-type (GG) than mutant type (GA + AA) [RR = 0.90, 95%CI (0.83, 0.98), *P* = 0.02], and the difference was statistically significant. (2) Grade 3–4: A total of six studies were included in the recessive gene model (GG vs. GA + AA), and the heterogeneity analysis showed that I^2^ = 25%, *P* = 0.25, and there was no statistical heterogeneity among the studies. The results showed that there was no significant difference in the incidence of grade 3–4 thrombocytopenia between UGT1A1*6 wild-type (GG) and mutant type (GA + AA) during IRI chemotherapy [RR = 1.14, 95%CI (0.44, 2.92), *P* = 0.79], as shown in Figure 6. In summary, the polymorphisms of UGT1A1*6 gene were associated with the incidence of mild thrombocytopenia and the incidence of wild-type (GG) in cancer patients during IRI chemotherapy.

**FIGURE 7:**
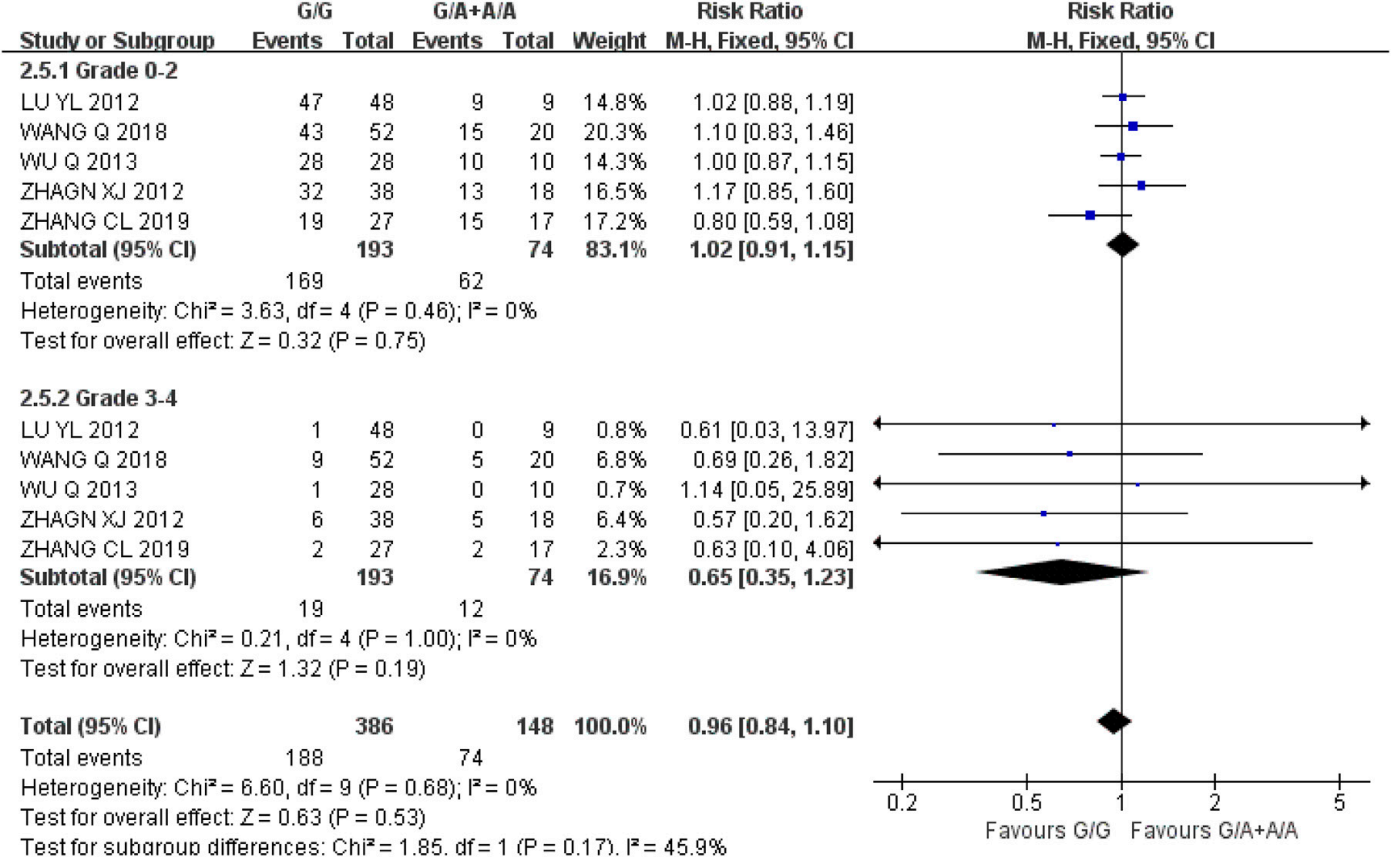
Forest map of Meta-analysis results of the correlation between UGT1A1*6 polymorphism and the incidence of hemoglobin.

*Hemoglobinia* Five studies were included in the recessive gene model (GG vs. GA + AA) at grades 0–2 and 3-4, and the heterogeneity analysis was I^2^ = 0%, *P* = 0.46, I^2^ = 0%, and *P* = 1.00, respectively, suggesting that there was no statistical heterogeneity between the two groups. The results showed that there was no significant difference in the incidence of hemoglobin reduction between UGT1A1*6 wild-type (GG) and mutant type (GA + AA) in grade 0–2 [RR = 1.02, 95%CI (0.91, 1.15), *P* = 0.75] and grade 3–4 [RR = 0.65, 95%CI (0.35, 1.23), *P* = 0.19] during IRI chemotherapy (*P* > 0.05), as shown in Figure 7. The results indicated that there was no correlation between UGT1A1*6 polymorphisms and hemoglobinia.

## Correlation of UGT1A1*28 polymorphism with efficacy and toxicity of IRI

### Efficacy evaluation

**FIGURE 8:**
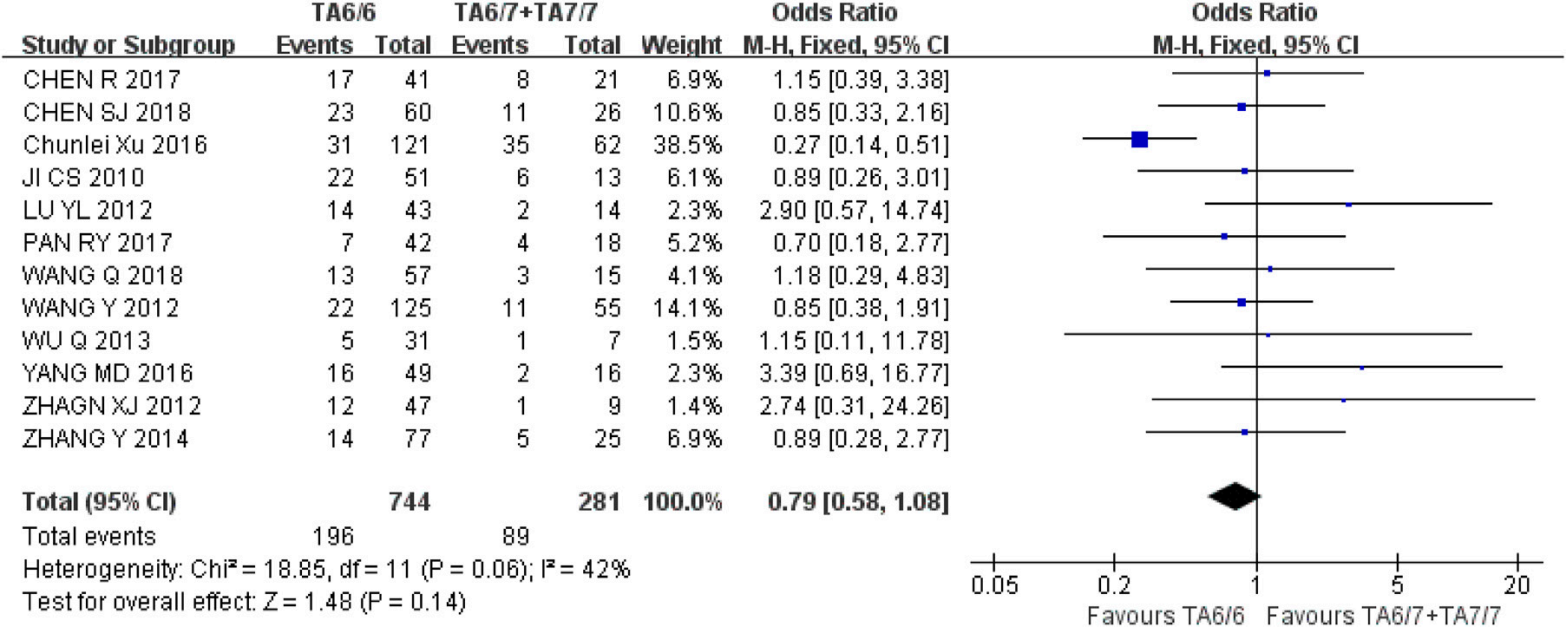
Forest map of Meta-analysis results of UGT1A1*28 and RR.

Similarly, disease response rate (RR = CR + PR) was selected to evaluate the relationship between UGT1A1*28 gene polymorphisms and IRI efficacy. Twelve studies with 1,025 people with cancer were included. We analysed the included studies using a recessive genetic model (TA6/6 vs. TA6/7 + TA7/7). Heterogeneity analysis showed that I^2^ = 42%, *P* = 0.06, and there was no statistical heterogeneity among the studies, and a fixed-effect model was used for analysis. The results showed that there was no significant difference in disease response rate [OR = 0.79, 95%CI (0.58, 1.08), *P* = 0.14] between UGT1A1*28 wild-type (TA6/6) and mutant type (TA6/7 + TA7/7) in the recessive gene model (*P* > 0.05), as shown in Figure 8. The results indicated that there was no correlation between UGT1A1*28 gene polymorphisms and IRI efficacy.

### Toxicity

**FIGURE 9:**
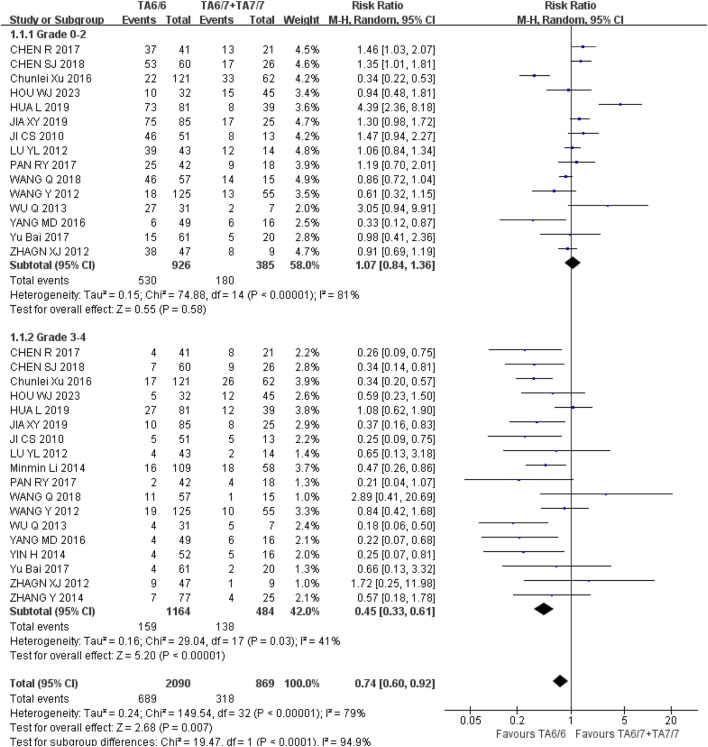
Forest map of Meta-analysis results of the correlation between UGT1A1*28 polymorphism and the incidence of diarrhea.

*Diarrhea* (1) Grade 0–2: A total of 15 studies were included in the recessive gene model (TA6/6 vs. TA6/7 + TA7/7), and the heterogeneity analysis showed that I^2^ = 81%, P < 0.00001, and there was statistical heterogeneity among the studies. The results showed that there was no statistically significant difference in the incidence of grade 0–2 diarrhea [RR = 1.07, 95%CI (0.84, 1.36), P = 0.58] between UGT1A1*28 wild-type (TA6/6) and mutant type (TA6/7 + TA7/7); (2) Grade 3–4: A total of 18 studies were included in the recessive gene model (TA6/6 vs. TA6/7 + TA7/7), and the heterogeneity analysis showed that I^2^ = 41%, *P* = 0.03, and there was no statistical heterogeneity among the studies. The results showed that UGT1A1*28 wild-type (TA6/6) had a lower incidence of grade 3–4 diarrhea than mutant type (TA6/7 + TA7/7) during IRI chemotherapy, and the difference was statistically significant [RR = 0.45, 95%CI (0.33, 0.61), *P* < 0.00001], as shown in Figure 9. Therefore, the polymorphisms of the UGT1A1*28 gene were associated with the incidence of grade 3–4 diarrhea in cancer patients during IRI chemotherapy, and the incidence of wild-type (TA6/6) was lower.

**FIGURE 10:**
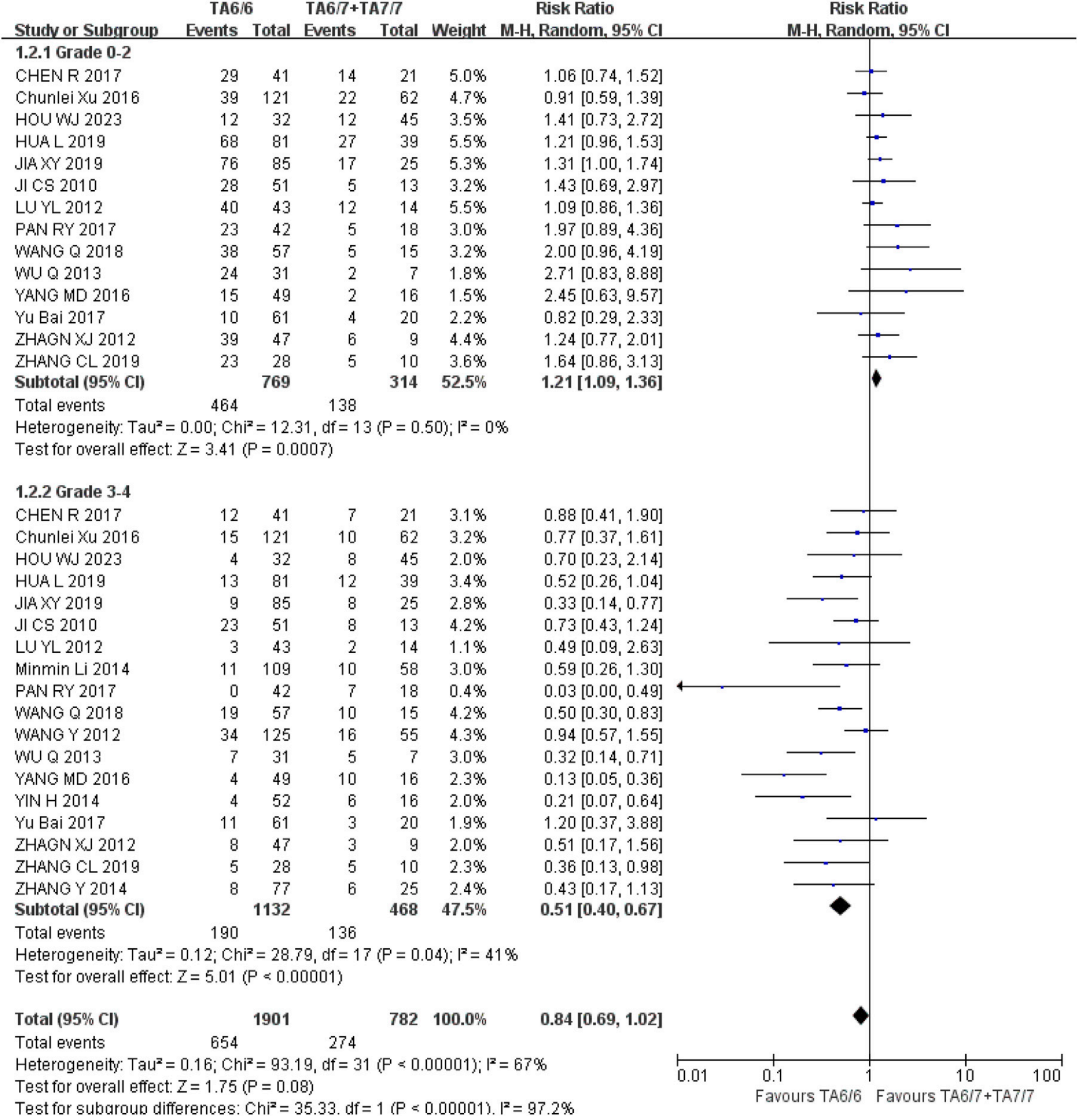
Forest map of Meta-analysis results of the correlation between UGT1A1*28 polymorphism and the incidence of granulocytopenia.

*Neutropenia* Grades 0–2 and 3–4: The recessive gene model (TA6/6 vs. TA6/7 + TA7/7) was included in 14 studies in both groups, with heterogeneity analyses of I^2^ = 0%, *P* = 0.50 and I^2^ = 41%, *P* = 0.04, respectively, with no statistical heterogeneity between studies. The results showed that the incidence of grade 0–2 and grade 3–4 neutropenia in the course of IRI chemotherapy was significantly different from that of UGT1A1*28 wild-type (TA6/6) compared with mutant type (TA6/7 + TA7/7), respectively [RR = 1.21, 95%CI (1.09, 1.36), P = 0.0007] and [RR = 0.51, 95%CI (0.40, 0.67), *P* < 0.00001], respectively, as shown in Figure 10. The above results suggested that the polymorphism of UGT1A1*28 gene was correlated with the incidence of neutropenia in cancer patients during IRI chemotherapy, and the incidence of wild-type (TA6/6) is higher in mild neutropenia, and the incidence of mutant type (TA6/7 + TA7/7) is higher in severe neutropenia.

**FIGURE 11:**
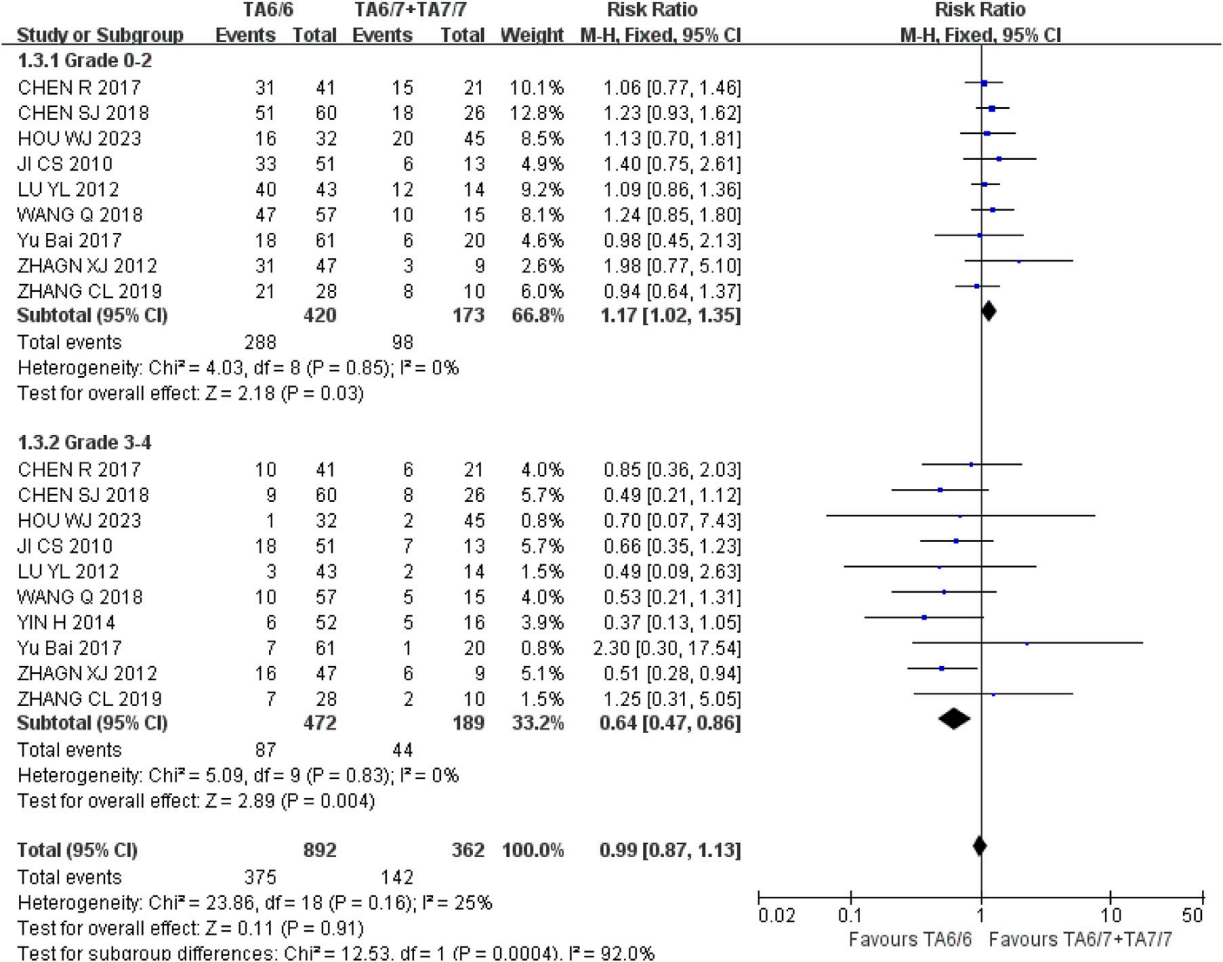
Forest map of Meta-analysis results of the correlation between UGT1A1*28 polymorphism and the incidence of leukopenia.

*Leukopenia* (1) Grade 0–2: A total of nine studies were included in the recessive gene model (TA6/6 vs. TA6/7 + TA7/7), and the heterogeneity analysis showed that it was I^2^ = 0%, *P* = 0.85, and there was no statistical heterogeneity among the studies. The results showed that UGT1A1*28 wild type (TA6/6) had a higher incidence of grade 0–2 leukopenia than mutant type (TA6/7 + TA7/7) [RR = 1.17, 95%CI (1.02, 1.35), *P* = 0.03], and the results were statistically different. (2) Grade 3–4: A total of 10 studies were included in the recessive gene model (TA6/6 vs. TA6/7 + TA7/7), and the heterogeneity analysis showed that I^2^ = 0%, *P* = 0.83, and there was no statistical heterogeneity among the studies. The results showed that UGT1A1*28 wild-type (TA6/6) had a statistically significant difference in the incidence of grade 3–4 leukopenia during IRI chemotherapy compared with mutant type (TA6/7 + TA7/7) [RR = 0.64, 95%CI (0.47, 0.86), *P* = 0.004], as shown in Figure 11. In summary, the polymorphisms of UGT1A1*28 gene were associated with the incidence of leukopenia in cancer patients during IRI chemotherapy, and the incidence of wild type (TA6/6) was higher in mild leukopenia, and the incidence of mutant type (TA6/7 + TA7/7) was higher in severe leukopenia.

**FIGURE 12:**
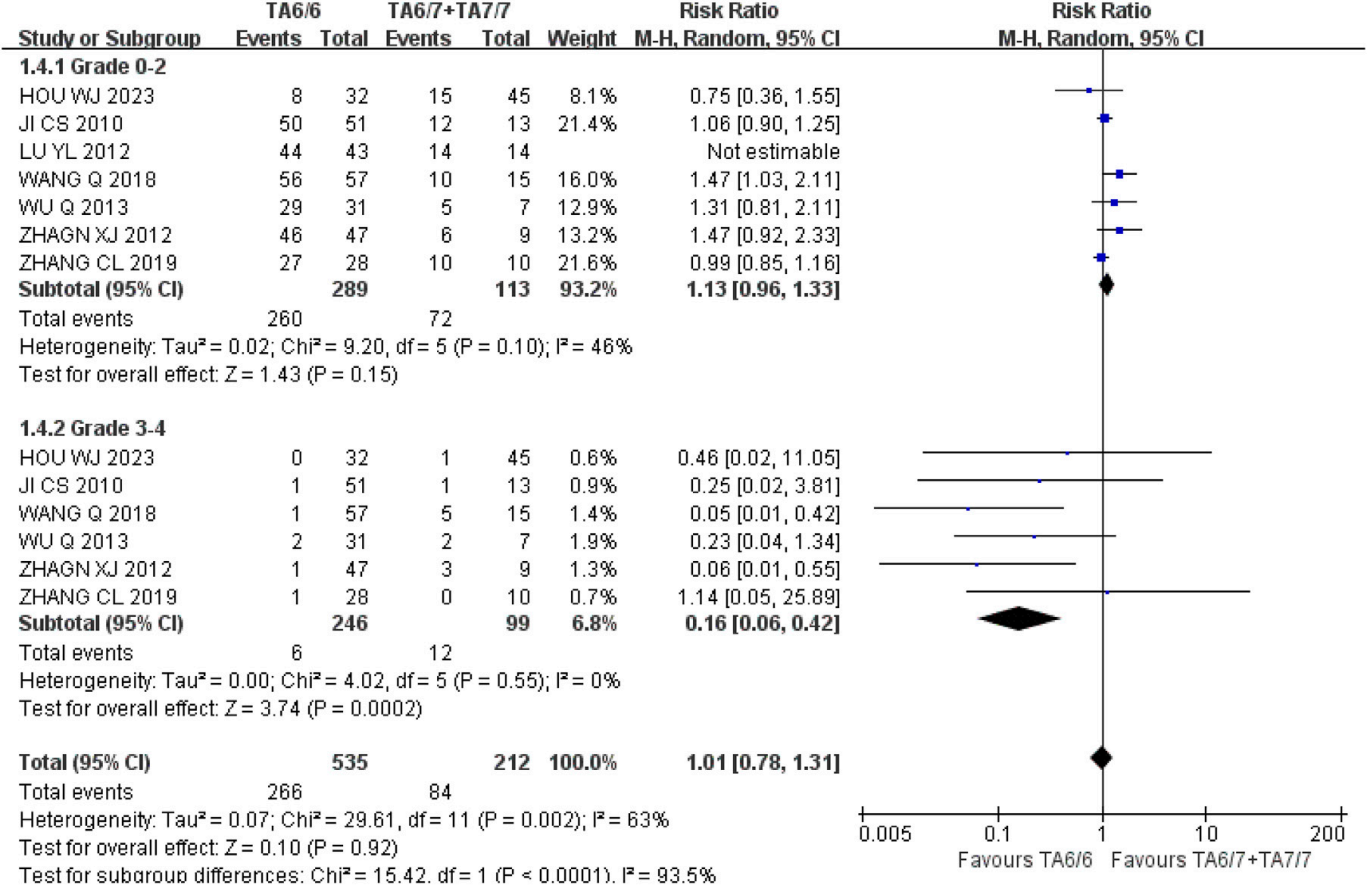
Forest map of Meta-analysis results of the correlation between UGT1A1*28 gene polymorphism and the incidence of thrombocytopenia.

*Thrombocytopenia* (1) Grade 0–2: A total of seven studies were included in the recessive gene model (TA6/6 vs. TA6/7 + TA7/7), and the heterogeneity analysis showed that it was I^2^ = 46%, *P* = 0.10, and there was no statistical heterogeneity among the studies. The results showed that UGT1A1*28 wild-type (TA6/6) had a higher incidence of grade 0–2 thrombocytopenia compared with mutant type (TA6/7 + TA7/7) [RR = 1.13, 95%CI (0.96, 1.33), *P* = 0.15], but there was no statistically significant difference in the results (*P* > 0.05); (2) Grade 3–4: A total of six studies were included in the recessive gene model (TA6/6 vs. TA6/7 + TA7/7), and the heterogeneity analysis showed that it was I^2^ = 0%, *P* = 0.55, and there was no statistical heterogeneity among the studies. The results showed that the incidence of grade 3–4 thrombocytopenia in the course of IRI chemotherapy was lower in UGT1A1*28 wild-type (TA6/6) than mutant type (TA6/7 + TA7/7), and the difference was statistically significant [RR = 0.16, 95%CI (0.06, 0.42), *P* = 0.0002], as shown in Figure 12. In summary, the polymorphism of UGT1A1*28 gene was associated with the incidence of severe thrombocytopenia in cancer patients during IRI chemotherapy, and the incidence of mutation (TA6/7 + TA7/7) was higher in severe thrombocytopenia.

**FIGURE 13:**
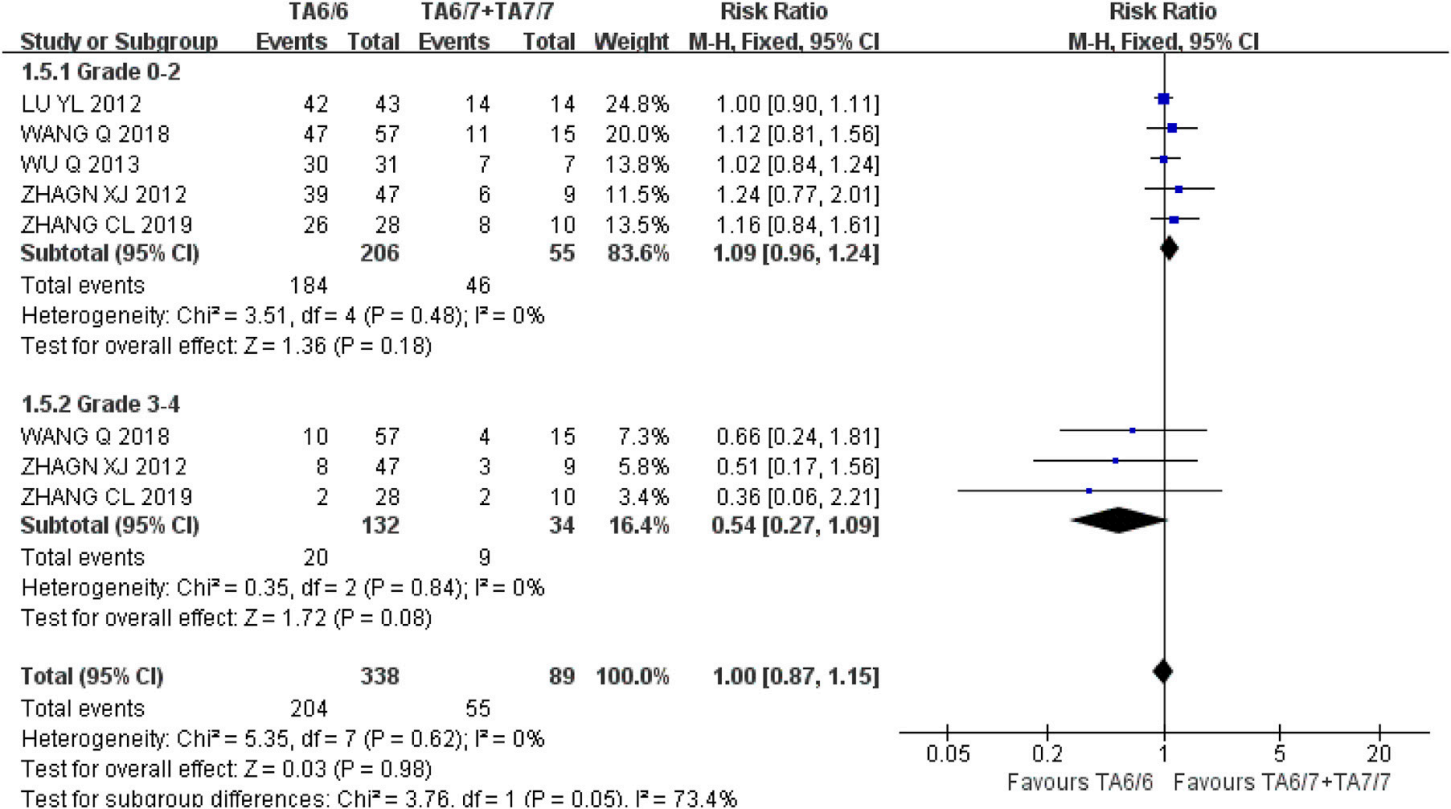
Forest map of Meta-analysis results of the correlation between UGT1A1*28 polymorphism and the incidence of hemoglobin.

*Hemoglobinia* At grades 0–2 and three to four, the recessive gene model (TA6/6 vs. TA6/7 + TA7/7) was included in five and three studies, respectively, and the heterogeneity analysis was I^2^ = 0%, *P* = 0.48, I^2^ = 0%, and *P* = 0.84, respectively, suggesting that there was no statistical heterogeneity between the two groups. The results showed that there was no significant difference in the incidence of grade 0–2 [RR = 1.09, 95%CI (0.96, 1.24), *P* = 0.18] and grade 3–4 [RR = 0.54, 95%CI (0.27, 1.09), *P* = 0.08] and grade 3–4 [RR = 0.54, 95%CI (0.27, 1.09), *P* = 0.08] in UGT1A1*28 wild-type (TA6/6) and mutant type (TA6/7 + TA7/7) during IRI chemotherapy (*P* > 0.05), as shown in Figure 13. The results indicated that there was no correlation between UGT1A1*28 gene polymorphisms and hemoglobin reduction.

## Correlation between UGT1A1*6 and UGT1A1*28 double-locus polymorphisms and the efficacy and toxicity of IRI

### Efficacy evaluation

The disease response rate (RR = CR + PR) was selected to evaluate the relationship between the polymorphism of UGT1A1*6 and UGT1A1*28 genes and the efficacy of IRI. Three studies were included, with 210 patients with cancer. We used double wild type (DW), single variant (SV) and double variant (DV) to analyze the included studies, respectively. The results showed that the disease remission rate of DW compared with SV during IRI chemotherapy for cancer [OR = 1.65, 95% CI (0.89, 3.05), *P* = 0.11], the disease remission rate of DW compared with DV [OR = 1.45, 95% CI (0.55, 3.84), *P* = 0.45] and the disease remission rate of SV compared with DV [OR = 0.56, 95% CI (0.21, 1.50), *P* = 0.25], none of the above three results were statistically significant (*P* > 0.05), suggesting that UGT1A1*6 and UGT1A1*28 dual sites have no correlation with IRI efficacy, as shown in Table 2.

**TABLE 2:** Meta-analysis results of the clinical efficacy and toxicity of UGT1A1*6 and UGT1A1*28 gene polymorphism in IRI treatment of tumors.

Clinical efficacy and toxicity	DW vs. SV		DW vs. DV		SV vs. DV	
	*OR (95%CI)*	*P*	*OR (95%CI)*	*P*	*OR (95%CI)*	*P*
RR	1.65 [0.89, 3.05]	0.11	1.45 [0.55, 3.84]	0.45	0.56 [0.21, 1.50]	0.25
Grade 0–2 diarrhea	0.60 [0.27, 1.37]	0.23	0.78 [0.16, 3.77]	0.76	1.58 [1.15, 2.18]	0.005
Grade 3–4 diarrhea	0.48 [0.30, 0.78]	0.003	0.14 [0.07, 0.25]	<0.00001	0.33 [0.19, 0.57]	<0.0001
Grade 0–2 neutropenia	0.93 [0.56, 1.54]	0.77	1.50 [1.03, 2.19]	0.03	1.81 [1.00, 3.29]	0.05
Grade 3–4 neutropenia	1.13 [0.76, 1.69]	0.54	0.46 [0.31, 0.69]	0.0002	0.36 [0.23, 0.57]	<0.0001
Grade 0–2 leukopenia	1.04 [0.76, 1.43]	0.81	1.95 [0.60, 6.37]	0.27	2.01 [0.60, 6.69]	0.26
Grade 3–4 leukopenia	0.94 [0.45, 1.98]	0.88	1.09 [0.25, 4.72]	0.91	0.89 [0.21, 3.78]	0.88

Notes: RR, disease remission rate; DW, double wild type; SV, single variant; DV, double variant.

### Toxicity

*Diarrhea* (1) Grades 0–2: four studies were included. Meta-analysis showed that the incidence of grade 0 to two diarrhea in DW compared with SV during IRI chemotherapy for cancer was [RR = 0.60, 95% CI (0.27, 1.37), *P* = 0.23]; The incidence of grade 0–2 diarrhea in DW compared with DV was [RR = 0.78, 95%CI (0.16, 3.77), *P* = 0.76]; The incidence of grade 0 to two diarrhea in SV compared with DV was [RR = 1.58, 95%CI (1.15, 2.18), *P* = 0.005]; (2) Grades 3–4: Six studies were included. Meta-analysis showed that the incidence of grade 3–4 diarrhea in DW compared with SV during IRI chemotherapy was [RR = 0.48, 95% CI (0.30, 0.78), *P* = 0.003]; The incidence of grade 3–4 diarrhea in DW compared with DV was [RR = 0.14, 95% CI (0.07, 0.25), *P* < 0.00001]; The incidence of grade 3 to 4 diarrhea in SV compared with DV was [RR = 0.33, 95% CI (0.19, 0.57), *P* < 0.0001]. The above results suggest that SV are more likely to have mild diarrhea, while the incidence of severe diarrhea is DV > SV > DW, as shown in Table 2.

*Neutropenia* (1) Grades 0–2: four studies were included. Meta-analysis showed that the incidence of grade 0–2 neutropenia in DW compared with SV during IRI chemotherapy was [RR = 0.93, 95%CI (0.56, 1.54), *P* = 0.77]; The incidence of grade 0–2 neutropenia in DW compared with DV was [RR = 1.50, 95% CI (1.03, 2.19), *P* = 0.03]; The incidence of grade 0 to two neutropenia in SV compared with DV was [RR = 1.81, 95% CI (1.00–3.29), *P* = 0.05]; (2) Grade three to 4: six studies were included. Meta-analysis showed that the incidence of grade 3–4 neutropenia in DW compared with SV during IRI chemotherapy was [RR = 1.13, 95%CI (0.76, 1.69), *P* = 0.54]; The incidence of grade 3–4 neutropenia in DW compared with DV was [RR = 0.46, 95%CI (0.31, 0.69), *P* = 0.0002]; The incidence of grade 3–4 neutropenia in SV compared with DV was [RR = 0.36, 95% CI (0.23, 0.57), *P* < 0.0001]. These results suggested that DW was more likely to develop mild neutropenia, and DV are more likely to have severe neutropenia, as shown in Table 2.

*Leukopenia* Grades 0–2 and 3–4: two studies were included. The results of meta-analysis showed that there was no statistically significant difference in the incidence of leukopenia between DW and SV in grade 0–2 [RR = 1.04, 95%CI (0.76, 1.43), *P* = 0.81] and grade 3–4 [RR = 0.94, 95%CI (0.45, 1.98), *P* = 0.88]; There was no significant difference in the incidence of leukopenia between DW and DV in grade 0–2 [RR = 1.95, 95%CI (0.60, 6.37), *P* = 0.27] and grade 3–4 [RR = 1.09, 95%CI (0.25, 4.72), *P* = 0.91]; There was no statistically significant difference in the incidence of leukopenia between SV and DV in grade 0–2 [RR = 2.01, 95%CI (0.60, 6.69), P = 0.26] and grade 3–4 [RR = 0.89, 95%CI (0.21, 3.78), *P* = 0.88]. The above results suggested that double-locus polymorphisms of UGT1A1*6 and UGT1A1*28 are not correlated with leukopenia, as shown in Table 2.

## Sensitivity analysis

Due to the inclusion of 19 articles, the methodological quality of some clinical trials is not high. Therefore, the sensitivity analysis of the results of meta-analysis was carried out by removing one test each time and then re-statistic. There was no significant difference in the analysis results before and after the elimination of all studies, suggesting that all Meta-analysis results were stable.

## Publication bias analysis

**FIGURE 14:**
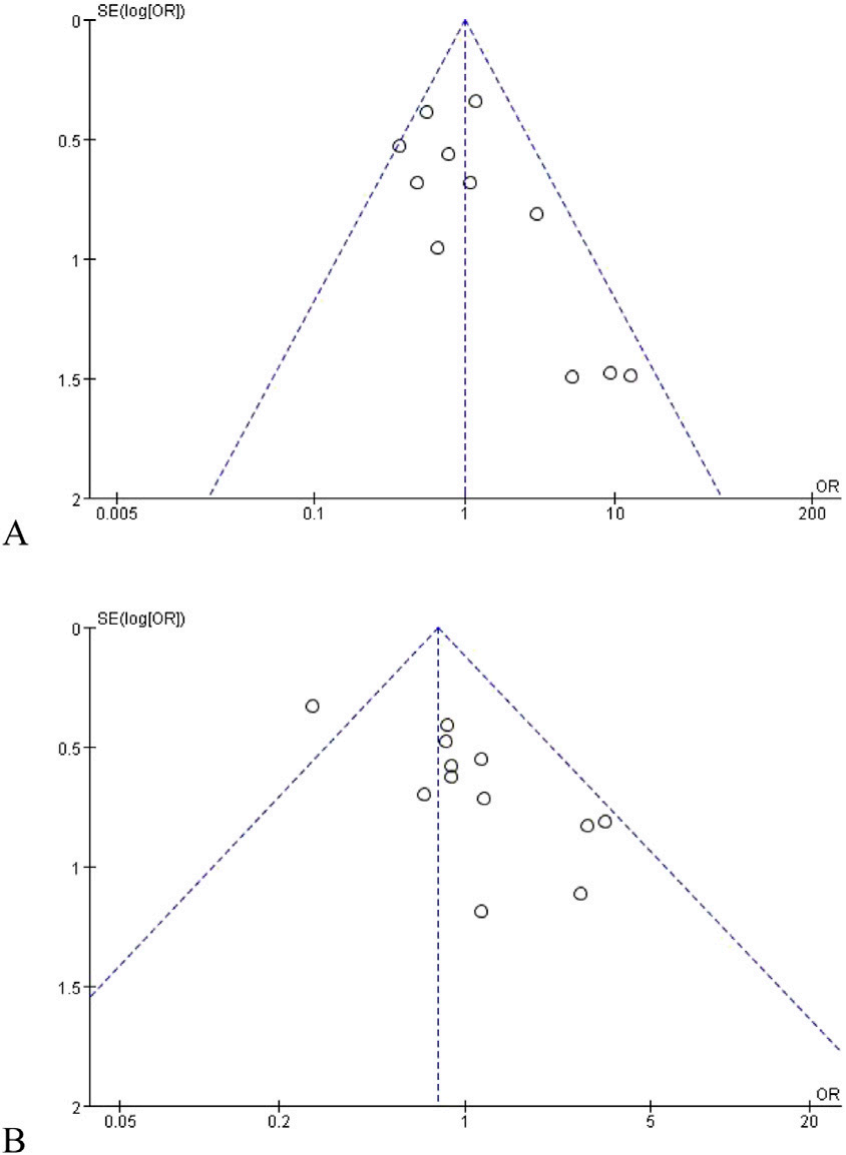
Rr funnel diagram of UGT1A1*6 **(A)** and UGT1A1*28 **(B)**.

When the number of included studies ≥10, the publication bias was tested by funnel plots, as recommended by the Cochrane Systematic Review Production Manual. We selected the disease response rates of UGT1A1*6 and UGT1A1*28 as indicators of publication bias for funnel plot statistics, and the results showed that the funnel graph was symmetrical, mainly concentrated in the middle and upper part, and only one study may be located on the outside of the inverted funnel plot due to factors such as poor design and poor research methods, and the overall result was no obvious publication bias, as shown in Figures 14A, B.

## Discussion

Irinotecan (IRI) is a prodrug, which is converted into the active metabolite 7-ethyl-10-hydroxycamptothecin (SN-38) *in vivo* by futinylase, and its activity is 100–1,000 times stronger than irinotecan. Uridine diphosphate glucuronosyltransferase 1A1 (UGT1A1), on the other hand, converts SN-38 into inactive SN-38 glucuronide (SN38G), which is excreted from the bile, thereby protecting healthy cells from irinotecan toxicity (Bailly, 2019).

Single nucleotide polymorphism (SNP) is widely present in the human genome and is the main form of heritable variation in the DNA sequence of the human genome, which refers to the polymorphism of the DNA sequence caused by the variation of a single nucleotide at the genomic level, which is related to ethnic diversity, disease susceptibility, and differences in drug response (Walter et al., 2008). The UGT1A1 gene is located on human chromosome 2q37, and the polymorphism of UGT1A1*6 manifests itself as a mutation in exon 1211G > A, forming three genotypes: wild-type (G/G), heterozygous mutant (A/G), and homozygous mutant (A/A), which can lead to an alteration of the amino acid sequence at position 71 of the UGT1A1 enzyme (Arg→Gly), resulting in a decrease in enzyme activity (Kane 2012). In addition, the promoter region of UGT1A1 gene had polymorphisms, and the TATA cassette region contained 5-8 TA repeats, and the expression of UGT1A1 decreased with the increase of the number of TA repeats. The general population has 6/6TA repeats, while a minority have 7/7 or 6/7TA duplicates (Goon et al., 2016).

Due to the differences in genetic background, the differences in UGT1A1 mutation sites and mutation frequencies are important reasons for the obvious differences in the efficacy and incidence of adverse reactions between different cancer populations in Eastern and Western countries when receiving IRI treatment. Studies have shown that (Hirose et al.,2012) UGT1A1*28 has a mutation rate of only 1.2%–5.0% in Asians, which is much lower than in Africans (12%–27%) and Caucasians (5%–15%). Other studies (Etienne et al., 2015) have shown that UGT1A1* 28 mutation rates are 38%–45% and 29%–39% in the Americas and Caucasians, respectively, while UGT1A1*28 mutation rates are about 15%–18% and homozygous mutations are about 3% in Asian races. The US Food and Drug Administration (FDA) has included a risk warning of the possibility of severe neutropenia following IRI chemotherapy in the drug description. Although there are many studies, there are still different opinions on whether UGT1A1 can predict its chemotherapy efficacy and toxicity. Therefore, this article uses meta-analysis to systematically evaluate the relationship between UGT1A1*6 and UGT1A1*28 polymorphisms and IRI efficacy and related toxicity, so as to provide more reliable evidence-based medical evidence for clinical treatment.

In this in-depth study, we systematically analyzed the impact of UGT1A1*6 and UGT1A1*28 polymorphisms on the efficacy and toxicity of irinotecan (IRI) chemotherapy in Chinese cancer patients. The study employed a meta-analysis approach to comprehensively assess multiple research findings, aiming to provide a scientific basis for personalized treatment in Chinese cancer patients. In terms of efficacy assessment, our results showed that under the recessive genetic model, individuals carrying UGT1A1*6, UGT1A1*28, or both variations (double variants) did not exhibit significant differences in response rate (RR). This finding suggests that UGT1A1 gene polymorphisms cannot effectively predict the efficacy of IRI chemotherapy in the Chinese cancer patient population. This conclusion is consistent with previous research by Chen et al. (2017b), further reinforcing the view that UGT1A1 polymorphisms are not directly associated with IRI efficacy. In terms of toxicity analysis, we found that the standard dose of IRI (180 mg/m^2^) may be insufficient to achieve the desired therapeutic effect for patients with wild-type and heterozygous mutations. Therefore, we recommend conducting genetic typing before initiating chemotherapy to guide the exploration of personalized dosing (Zhou CF et al., 2013). Specifically, for patients carrying the wild-type alleles of UGT1A1*28 and UGT1A1*6, a moderate increase in the IRI dose may lead to better clinical outcomes without significantly increasing the risk of diarrhea and hematological toxicity. In fact, previous studies (Etienne-Grimaldi MC et al., 2015) have determined the maximum tolerated dose of IRI under a FOLFIRI regimen guided by genetic typing, with 420 mg/m^2^ for patients with the 6/6 wild-type and 370 mg/m^2^ for those with the 6/7 variant. However, it is worth noting that the IRI doses in the studies included in this meta-analysis generally remained at the standard dose level (180 mg/m^2^), administered once daily *via* intravenous infusion. Furthermore, our study revealed associations between UGT1A1*6 and UGT1A1*28 polymorphisms and various severe toxic events during IRI chemotherapy. Specifically, these polymorphisms are associated with an increased risk of grade 3–4 diarrhea, grade 3–4 neutropenia, grade 3–4 leukopenia, and grade 3–4 thrombocytopenia. However, the incidence of these toxic reactions is relatively low in patients with the wild-type alleles (GG and TA6/6). In particular, patients with the UGT1A1*6 wild-type (GG) have a lower incidence of grade 0–2 thrombocytopenia but a higher incidence of grade 0–2 neutropenia. In contrast, patients with the UGT1A1*28 wild-type (TA6/6) have a higher incidence of grade 0–2 neutropenia and grade 0–2 leukopenia. Additionally, we observed that among patients with double variants (DV) and IRI toxicity, those with double wild-type alleles (DW) are more prone to grade 0–2 neutropenia, those with single variants (SV) are more prone to grade 0–2 diarrhea, and those with double variants (DV) are more prone to grade 3–4 neutropenia. However, these polymorphisms are not directly associated with the occurrence of leukopenia. Among all the analysis results, we also found a correlation between UGT1A1 polymorphisms and hemoglobin reduction. In summary, this study demonstrates that UGT1A1*6 and UGT1A1*28 polymorphisms can serve as important molecular markers for predicting varying degrees of toxicity induced by IRI chemotherapy in Chinese cancer patients, particularly in predicting severe toxic events such as severe diarrhea and severe neutropenia. Meanwhile, these polymorphisms are also associated with the incidence of leukopenia and thrombocytopenia, providing a scientific basis for formulating personalized treatment plans in clinical practice. In the future, with the continuous development of precision medicine, individualized chemotherapy strategies based on genetic typing are expected to further improve the treatment effect and quality of life of cancer patients.

In exploring the relationship between UGT1A16 and UGT1A128 polymorphisms and the efficacy of irinotecan (IRI), this study indeed encountered and identified several limitations that need to be addressed and improved in future research: (1) Publication Bias Due to Incomplete Literature Coverage: During the literature search process, this study may have overlooked some conference papers, gray literature (such as unpublished research reports, dissertations, etc.), and other relevant literature. These documents may contain important research data and conclusions. This incomplete coverage of literature may lead to publication bias, where certain types or results of studies are not included, affecting the representativeness of the overall conclusion. (2) Geographical Limitation and Publication Bias: All the original literature included in this study originated from China. This geographical limitation may question the universality and applicability of the research results. Domestic studies may be influenced by factors such as specific medical environments, patient population characteristics, and treatment options, making it difficult to generalize the research conclusions globally. Therefore, this geographical limitation may also lead to publication bias, as domestic research findings may not fully reflect the global situation. (3) Limitation in Genetic Model Analysis: Due to the constraints of the original literature data, this study only conducted an in-depth analysis of the recessive genetic model. However, the impact of genetic polymorphisms on drug response often involves complex interactions among multiple genetic models. Therefore, analyzing only the recessive genetic model may not fully reveal the complete relationship between UGT1A16 and UGT1A128 polymorphisms and the efficacy and adverse reactions of irinotecan. Future research should further expand to other genetic models to provide a more comprehensive assessment of genetic effects. (4) Lack of Analysis on Drug Dosage Effects: Drug dosage is one of the key factors affecting drug efficacy and adverse reactions. However, due to the limitations of the original literature data, this study did not conduct an in-depth analysis of adverse reactions caused by drug dosage. This limits our comprehensive understanding of the efficacy and safety of irinotecan at different doses. Future research should strengthen the exploration of drug dosage effects to guide individualized adjustments in clinical medication. (5) Challenge of Heterogeneity Among Studies: Differences in research methods, sample selection, data analysis, and other aspects among studies may lead to inconsistency or heterogeneity in research results. This heterogeneity increases the difficulty of interpreting the results and may weaken the reliability of the research conclusions. To overcome this challenge, future research should adopt uniform research designs and analytical methods as much as possible and strengthen international cooperation and exchange to form more consistent and reliable research conclusions.

In summary, although this study revealed a close relationship between UGT1A16 and UGT1A128 polymorphisms and irinotecan toxicity, it did not find a significant difference in short-term efficacy. This conclusion is limited by the number and quality of the studies included. To enhance the universality and reliability of the results, future research should consider incorporating more international data to expand the sample size and diversify research methods. At the same time, in-depth analysis of genetic models, drug dosage effects, and heterogeneity among studies should be strengthened to promote the in-depth development of research in this field.

## Data Availability

The original contributions presented in the study are included in the article/supplementary material, further inquiries can be directed to the corresponding author.

## Abbreviations

CR, Complete response; DV, Double variant; DW, Double wild type; FDA, Food and Drug Administration; IRI, Irinotecan; OR, Odds ratio; PR, Partial response; PD, Progressive disease; RR, Relative risk; SN-38G, Glycosylated SN-38; SNP, Single nucleotide polymorphism; SD, Stable disease; SV, Single variant; UGT1A1, Uridine diphosphate glucuronide transferase 1A1; NOS, Newcastle-Ottawa scale.
